# Tolerability of COVID-19 mRNA vaccines in patients with postural tachycardia syndrome: a cross-sectional study

**DOI:** 10.12688/f1000research.109373.1

**Published:** 2022-02-22

**Authors:** Karin Jost, Belén Rodriguez, Nicole Söll, Robert Hoepner, Werner J. Z'Graggen

**Affiliations:** 1 Department of Neurology, Inselspital, University Hospital Bern, Bern, Bern, 3010, Switzerland; 2 Department of Neurosurgery, Inselspital, University Hospital Bern, Bern, Bern, 3010, Switzerland

**Keywords:** autonomic dysfunction, orthostatic intolerance, autoimmune, autonomic neuropathy

## Abstract

**Background:** Postural tachycardia syndrome (POTS) is a form of autonomic dysregulation. There is increasing evidence that the etiology may be immune-mediated in a subgroup of patients. Patients with POTS often experience an exacerbation of their symptoms associated with (viral) infections and often fear the same symptom aggravation after vaccination. In this report we describe the tolerability of messenger ribonucleic acid (mRNA) vaccines against coronavirus disease 19 (COVID-19) and the consequences of a COVID-19 infection on POTS symptoms in our cohort of patients with neuropathic POTS.

**Methods:** We conducted a standardized, checklist-based interview with 23 patients and recorded the acute side effects of mRNA vaccination, acute symptoms of COVID-19 infection as well as the effects of vaccination and COVID-19 infection on POTS symptoms.

**Results**: Of all included patients, 20 patients received two mRNA vaccines without having had a previous COVID-19 infection, and five patients in total had suffered a COVID-19 infection. Of these, three had COVID-19 without and two after being vaccinated. No increased frequency of side effects after both doses of mRNA vaccines was observed. Six patients reported a mild and short-term aggravation of their POTS symptoms beyond the duration of acute vaccine side effects. All five patients who suffered a COVID-19 infection subsequently reported a pronounced and persistent exacerbation of POTS symptoms.

**Conclusions:** Our observations suggest that mRNA vaccines are not associated with a higher frequency of acute side effects in patients with POTS. Symptom exacerbation as a consequence of mRNA vaccination seems to be less frequent and of shorter duration compared to patients who suffered a COVID-19 infection.

## Introduction

Postural tachycardia syndrome (POTS) results from autonomic dysregulation. It is characterized in adults by a clinically symptomatic, sustained increase in heart rate of more than 30 beats per minute within 10 minutes of standing or head-up tilt testing, in the absence of orthostatic hypotension. ^1^^,^^2^ Patients with POTS experience symptoms of orthostatic intolerance in the upright position such as lightheadedness, dizziness, palpitations, tremulousness, generalized weakness and leg pain, blurred vision, dyspnea, nausea, headache and cognitive dysfunction. ^3^^–^^6^ Many patients with POTS additionally report non-orthostatic symptoms of autonomic origin such as fatigue, gastrointestinal complaints, sleep disturbances, restless legs symptoms and exercise intolerance. ^7^^,^^8^ The exact etiology of POTS is still unknown, although in recent years evidence has accumulated that in a subset of patients with POTS the pathogenesis of dysautonomia may be immune-mediated. ^9^^,^^10^ The onset of POTS is frequently reported after an immunologic stressor, with a female predominance. ^11^^,^^12^ Up to 50% of patients with POTS describe a viral infection as the trigger of their symptoms. ^12^^,^^13^ Patients also often report that infections (especially viral illnesses) are triggers for a prolonged symptom exacerbation, even after the subsiding of the acute infection. ^4^^,^^13^^,^^14^ Individuals with POTS are more likely to be affected by comorbid autoimmune diseases than the average population. ^8^^,^^15^^,^^16^ In recent years, autoantibodies against G-coupled protein receptors, most often including autoantibodies against adrenergic and cholinergic receptors, were characterized in POTS. ^9^^,^^17^^–^^19^ Autoantibodies against the α1-adrenergic receptor were the most common among them. ^18^^–^^20^ Additionally, antibodies against angiotensin II type 1 receptors and abnormal levels of inflammatory biomarkers were reported. ^21^^,^^22^ Despite the presence of these antibodies, their role in the complex pathophysiology of autonomic dysfunction in POTS remains unknown. ^15^ Immunomodulatory treatment with intravenous immunoglobulins has shown a positive effect on the symptoms of patients with POTS, further supporting an immune-mediated genesis. ^23^

In the wake of the coronavirus disease 2019 (COVID-19) pandemic, numerous case reports and case series about the occurrence of POTS following an infection with the severe acute respiratory syndrome coronavirus 2 (SARS-CoV-2) have accumulated. ^24^^–^^30^ Nearly all affected individuals were females without pre-existing conditions who developed symptoms of autonomic dysfunction several days or weeks after an acute COVID-19 infection and there was no association with initial COVID-19 severity. ^24^^,^^31^^,^^32^ Vaccines based on messenger ribonucleic acid (mRNA) technology are being used to combat the COVID-19 pandemic. The mRNA provides the body with the genetic code of the virus, which is then translated in the host cells and as a consequence, spike proteins are built. These act as antigens and trigger an immune response, as a result of which neutralizing antibodies against SARS-CoV-2 are formed. ^33^ There is one case report in which POTS was diagnosed in a previously healthy, 42-year-old male following the first dose of mRNA vaccination. ^34^

We have observed that patients with POTS are hesitant towards vaccination in general and especially towards the new mRNA vaccines because they often fear aggravation of their symptoms. On the other side, it is reasonable to assume that a COVID-19 infection in patients with POTS may trigger a prolonged symptom amplification as it is commonly observed with infections.

The aim of this study was to assess the tolerability and side effects of the two COVID-19 mRNA vaccines used in Switzerland (Spikevax ^®^, Moderna; BNT162b2 ^®^, Pfizer) in a cohort of patients with POTS, and to assess possible consequences of a COVID-19 infection on POTS symptoms.

## Methods

### Patients

We conducted a standardized checklist-based interview with all patients who had been diagnosed with neuropathic POTS and were followed in the Autonomic Unit of the Departments of Neurology and Neurosurgery, University Hospital Bern, Bern, Switzerland. All available patients were contacted by telephone and asked if they were interested in participating in the study after checking the eligibility criteria. If interested, they were sent the informed consent form. After receiving the signed consent form the interview took place. The structured interviews were performed by one of two authors (KJ or BR) either by telephone or during a routine consultation. Data was collected between November 2021 and January 2022. All contacted patients agreed to participate in the study and provided written informed consent for the collection and publication of their data. Potential bias was minimized by the standardization and structuring of the interview. The interviewer strictly followed the predetermined interview checklist (please see the extended data for the used interview checklist). ^48^ The study was carried out in accordance with the Declaration of Helsinki. The diagnosis of POTS had been made according to medical history, physical and neurological examination, cardiovascular autonomic function testing, thermoregulatory sweat test and/or quantitative testing of sudomotor axon reflex, determination of autoantibodies against G-protein-coupled receptors, measurement of plasma norepinephrine levels and skin biopsy in selected patients. ^1^^,^^35^

### Eligibility criteria

Patients had to meet the following inclusion criteria: confirmed diagnosis of neuropathic POTS, aged between 18 and 60 years, received two COVID-19 mRNA vaccine doses ≥ 1 month prior to the interview, or recovered from COVID-19 infection ≥ 1 month prior to the interview.

### Interview checklists

To evaluate the tolerability of COVID-19 mRNA vaccines, the following data were collected during the interviews: Date(s) of vaccination and type of vaccine (BNT162b2 ^®^, Pfizer BioNTech, New York, NY or Spikevax ^®^, Moderna, Cambridge, MA). The following, previously published side effects of vaccines ^36^^–^^38^ were assessed (for the first and second dose of the vaccine separately) in their presence (yes/no) and duration (days): fever, shivering, fatigue, headache, joint pain, muscle pain, nausea, emesis, diarrhea, and reaction at injection site (pain, swelling and cutaneous reaction). ^49^

In patients who had suffered a COVID-19 infection, the following additional symptoms were queried: coughing, sore throat, rhinorrhea, breathlessness, loss of taste, loss of smell and chest pain. For each symptom, the presence (yes/no), severity (mild, moderate, severe) and duration (in days) were evaluated. Furthermore, the duration of the infection, need for hospitalization and incapacity for work were assessed.

To assess possible exacerbation of POTS symptoms due to mRNA vaccination and COVID-19 infection, the presence (yes/no), severity (mild, moderate, severe; for COVID-19 infection only) and duration of symptom exacerbation (in days) for the following symptoms were evaluated: dizziness, nausea, weakness, palpitations, lightheadedness, tremulousness, blurred vision, concentration difficulties, memory difficulties, orthostatic leg and/or arm pain, gastrointestinal symptoms, sleep disturbances, restless legs syndrome and orthostatic headache. During the interview, symptom aggravation was assessed separately for the first and second dose of the vaccine, and COVID-19 infection, from the patients memory. Furthermore, adjustment of therapy and inability for work due to symptom exacerbation were assessed.

### Data analysis

The data analysis was descriptive and performed using Statistical Package for the Social Sciences (SPSS Statistics) Version 25.0 (IBM Corp., Armonk, NY, USA). Data are reported either as frequencies, mean (range) or median (range). All interviews were fully completed, so there were no missing data.

## Results

### Patients

A total of 23 patients, two men (8.7%) and 21 women (91.3 %) with diagnosed neuropathic POTS and a mean age of 26.65 (range 18-40) years, were included in this study and interviewed once. In total, 20 patients who had been vaccinated twice and had not previously suffered a COVID-19 infection were assessed for side effects of the vaccinations. Of the 23 patients included in this study five (21.7%) had suffered a COVID-19 infection; three before and two after two doses of mRNA vaccination. ^48^

### Acute side effects of mRNA vaccination

Frequencies of published acute side effects of mRNA vaccination reported by our POTS cohort are shown in Table 1. All included patients received the first dose between April and September 2021 and the second dose between May and October 2021. After the first dose, patients were unable to work for a mean of 0.35 (range 0-7) days and after the second dose for a mean of 1.05 (range 0-3) days. No allergic reactions were observed.

**Table 1.** Acute side effects of messenger ribonucleic acid vaccination.

		First vaccination	Second vaccination
**Number** N		20	20
**Type of vaccine** N (%)			
BNT162b2 ^®^, Pfizer BioNTech		7 (35)	7 (35)
Spikevax ^®^, Moderna		13 (65)	13 (65)
**Side effect**			
**Fever**	**Presence** N (%)	4 (20)	13 (65)
	**Duration** median (range)	1 (1-2)	2 (1-4)
**Shivering**	**Presence** N (%)	3 (15)	13 (65)
	**Duration** median (range)	1 (1-2)	1 (1-4)
**Fatigue**	**Presence** N (%)	10 (50)	16 (80)
	**Duration** median (range)	2.5 (1-14)	2.5 (1-14)
**Headache**	**Presence** N (%)	6 (30)	15 (75)
	**Duration** median (range)	4 (1-5)	2 (1-14)
**Joint pain**	**Presence** N (%)	3 (15)	6 (30)
	**Duration** median (range)	3 (1-7)	2 (1-4)
**Muscle pain**	**Presence** N (%)	5 (25)	9 (45)
	**Duration** median (range)	3 (2-7)	2 (1-4)
**Nausea**	**Presence** N (%)	3 (15)	5 (25)
	**Duration** median (range)	7 (2-14)	2 (1-14)
**Emesis**	**Presence** N (%)	0	0
	**Duration** median (range)	0	0
**Diarrhea**	**Presence** N (%)	0	1 (5)
	**Duration** median (range)	0	3
**Reaction at injection site: pain**	**Presence** N (%)	14 (70)	17 (85)
	**Duration** median (range)	2 (1-4)	2 (1-5)
**Reaction at injection site: swelling and cutaneous reaction**	**Presence** N (%)	4 (20)	1 (5)
	**Duration** median (range)	3.5 (1-42)	42

Duration is given in days.

### Acute symptoms of COVID-19 infection

Acute symptoms of COVID-19 infection are summarized in Table 2. Mean duration of infection was 16.4 (range 10 – 27) days. None of the patients had to be hospitalized. Mean duration of incapacity for work was 18.8 (range 10 – 28) days.

**Table 2.** Acute symptoms of COVID-19 infection.

		Patient 1	Patient 2	Patient 3	Patient 4 *	Patient 5 *
**Age**		22	28	27	24	26
**Sex**		Male	Female	Female	Female	Female
**Symptom**						
**Fever**	**Presence**	Severe	No	Moderate	No	Moderate
	**Duration**	18 days	-	4 days	-	5 days
**Shivering**	**Presence**	Severe	No	Moderate	Moderate	No
	**Duration**	7 days	-	4 days	3 days	-
**Fatigue**	**Presence**	Severe	Severe	Severe	Severe	Severe
	**Duration**	1 month	20 days	21 days	18 days	14 days
**Headache**	**Presence**	Severe	Mild	Moderate	Moderate	Moderate
	**Duration**	14 days	14 days	21 days	4 days	6 days
**Joint pain**	**Presence**	No	Mild	Moderate	Moderate	Severe
	**Duration**	-	4 days	21 days	5 days	3 days
**Muscle pain**	**Presence**	Moderate	Mild	Moderate	Moderate	No
	**Duration**	14 days	4 days	21 days	5 days	-
**Nausea**	**Presence**	Severe	No	Moderate	Moderate	Severe
	**Duration**	1 month	-	12 days	7 days	3 days
**Emesis**	**Presence**	Severe	No	No	Mild	No
	**Duration**	1 month	-	-	1 day	-
**Diarrhea**	**Presence**	Moderate	No	Severe	Moderate	Moderate
	**Duration**	4 days	-	18 days	6 days	3 days
**Coughing**	**Presence**	Moderate	Mild	Moderate	Mild	No
	**Duration**	14 days	2 days	21 days	6 days	-
**Sore throat**	**Presence**	Moderate	Severe	Moderate	Moderate	No
	**Duration**	7 days	2 days	21 days	7 days	-
**Rhinorrhea**	**Presence**	No	Mild	Mild	Severe	Moderate
	**Duration**	-	2 days	6 days	7 days	5 days
**Breathlessness**	**Presence**	Severe	No	Moderate	No	Moderate
	**Duration**	14 days	-	21 days	-	5 days
**Loss of taste**	**Presence**	Severe	No	Severe	Mild	No
	**Duration**	3 months	-	5 months	1 day	-
**Loss of smell**	**Presence**	Moderate	Severe	Moderate	No	No
	**Duration**	3 months	1 month	2 months	-	-
**Chest pain**	**Presence**	No	No	Mild	No	Moderate
	**Duration**	-	-	21 days	-	5 days

* COVID-19 infection after 2 doses of mRNA vaccine (Spikevax ^®^, Moderna).

### Effect of mRNA vaccination on POTS symptoms

Reported increase of POTS symptoms after mRNA vaccination is shown in Table 3. An increase of POTS symptoms was reported by three patients after the first and by five patients after the second vaccination. Mean duration of symptom increase was seven days (range 1-14). None of the patients needed an adjustment of the symptomatic therapy for POTS, and no incapacity for work was reported.

**Table 3.** Effect of mRNA vaccination on POTS symptoms.

		First vaccination	Second vaccination
**Number** N		20	20
**Symptom increase**			
**Dizziness**	**Presence** N (%)	-	2 (10)
	**Duration** median (range)	-	6.5 (3-10)
**Weakness**	**Presence** N (%)	2 (10)	4 (20)
	**Duration** median (range)	9.5 (5-14)	6.5 (3-14)
**Lightheadedness**	**Presence** N (%)	-	2 (10)
	**Duration** median (range)	-	6.5 (3-10)
**Tremulousness**	**Presence** N (%)	-	1 (5)
	**Duration** median (range)	-	3
**Blurred vision**	**Presence** N (%)	-	1 (5)
	**Duration** median (range)	-	10
**Concentration difficulties**	**Presence** N (%)	1 (5)	2 (10)
	**Duration** median (range)	14	8.5 (3-14)
**Orthostatic arm and/or leg pain**	**Presence** N (%)	1 (5)	2 (10)
	**Duration** median (range)	5	4 (1-7)
**Restless legs syndrome**	**Presence** N (%)	-	1 (5)
	**Duration** median (range)	-	7
**Orthostatic headache**	**Presence** N (%)	2 (10)	1 (5)
	**Duration** median (range)	6 (5-7)	3

Duration is given in days. Only symptoms reported by at least one patient are listed.

### Consequences of COVID-19 infection regarding POTS symptoms

The effects of COVID-19 infection on POTS symptoms are shown in Table 4. In addition to the above reported incapacity for work, one patient (Patient 3) had to reduce her existing workload for two more months. Adjustment of symptomatic POTS treatment was necessary in all patients.

**Table 4.** Consequences of COVID-19 infection regarding POTS symptoms.

		Patient 1	Patient 2	Patient 3	Patient 4 *	Patient 5 *
**Age**		22	28	27	24	26
**Sex**		Male	Female	Female	Female	Female
**Symptom increase**						
**Dizziness**	**Presence**	Mild	Moderate	Mild	Moderate	Moderate
	**Duration**	Persistent	1 month	4 months	1 month	1 week
**Nausea**	**Presence**	No	No	No	No	Mild
	**Duration**	-	-	-	-	5 weeks
**Weakness**	**Presence**	Mild	Moderate	Moderate	Mild	Mild
	**Duration**	Persistent	1 month	4 months	1 month	5 weeks
**Palpitations**	**Presence**	No	Mild	Mild	No	No
	**Duration**	-	5 months	2 months	-	-
**Lightheadedness**	**Presence**	No	No	Mild	No	Mild
	**Duration**	-	-	6 months	-	5 weeks
**Tremulousness**	**Presence**	No	No	No	No	No
	**Duration**	-	-	-	-	-
**Blurred vision**	**Presence**	No	No	Mild	No	No
	**Duration**	-	-	6 months	-	-
**Concentration difficulties**	**Presence**	Severe	Moderate	Moderate	No	Mild
	**Duration**	Persistent	1 month	6 months	-	5 weeks
**Memory difficulties**	**Presence**	No	No	No	No	No
	**Duration**	-	-	-	-	-
**Orthostatic arm and/or leg pain**	**Presence**	Moderate	Moderate	Mild	Moderate	No
	**Duration**	Persistent	1 month	4 months	1 month	-
**Gastrointestinal symptoms**	**Presence**	No	No	Severe	Mild	Moderate
	**Duration**	-	-	3 months	1 month	5 weeks
**Sleep disturbances**	**Presence**	Severe	No	No	No	Moderate
	**Duration**	Persistent	-	-	-	5 weeks
**Restless legs syndrome**	**Presence**	Moderate	No	No	No	No
	**Duration**	Persistent	-	-	-	-
**Orthostatic headache**	**Presence**	Mild	No	Mild	No	No
	**Duration**	Persistent	-	Persistent	-	-

* COVID-19 infection after 2 doses of mRNA vaccine (Spikevax ^®^, Moderna).

## Discussion

The present study investigated the frequencies of known side effects of mRNA vaccination (Spikevax ^®^, Moderna; BNT162b2 ^®^, Pfizer) in patients with POTS. In addition, possible effects on POTS symptoms were assessed and compared to the impact of a COVID-19 infection.

Vaccine side effects were present in 20 (100%) patients for both vaccinations. The most frequently reported side effects of mRNA vaccines were pain at the injection site (70% after first, 85% after second), fatigue (50% after first, 80% after second), headache (30% after first, 75% after second), fever (20% after first, 65% after second) and shivering (15% after first, 65% after second). Side effects were generally reported more frequently after the second vaccination. This is in line with the results of other studies investigating the side effects of mRNA vaccines on healthy subjects as well as with the data from the vaccine manufacturers. ^33^^,^^36^^–^^38^

Only six patients reported mild worsening of their POTS symptoms after vaccination beyond the duration of the acute side effects, for a mean duration of seven days (maximum 14 days). The observed increase in symptoms occurred more frequently after the second vaccination. This is similar to findings of studies examining the effects of mRNA vaccination on disease activity in patients with autoimmune inflammatory rheumatic diseases, which showed no higher incidence of side effects compared to healthy subjects and no greater risk of disease flares. ^39^^,^^40^ Similarly, also patients who suffered from post-COVID symptoms of dysautonomia did not report a worsening of symptoms after getting vaccinated. ^41^

In contrast, patients suffering a COVID-19 infection experienced a pronounced and prolonged aggravation of their POTS symptoms for several months. Due to the symptom increase all patients needed an adjustment of their symptomatic POTS therapy and had prolonged incapability for work. Interestingly, symptom exacerbation due to a COVID-19 infection was also observed in two previously vaccinated patients. However, both patients were vaccinated more than six months prior to the infection at a time when booster vaccinations were not yet available for this priority group in Switzerland. In these two patients, there was a tendency for a milder and shorter exacerbation of POTS symptoms compared to non-vaccinated patients.

Most patients with POTS experience a prolonged increase of their symptoms in the context of infections (especially of viral etiology). ^4^^,^^13^^,^^14^ In general, hypovolemia, fever and bedrest can intensify POTS symptoms. ^26^^,^^42^^,^^43^ Furthermore, in patients with possible immune-mediated POTS, symptom aggravation is most likely due to a general immunological activation. Besides this, SARS-CoV-2 appears to affect the autonomic nervous system directly, which could be an additional factor for aggravation. ^44^ Since the onset of the COVID-19 pandemic, an increasing number of case reports about the occurrence of POTS secondary to a COVID-19 infection have emerged. ^24^^–^^30^ Several hypotheses about possible pathomechanisms of POTS or dysautonomia in general after COVID-19 infection have been proposed: imbalance of the renin-angiotensin-aldosterone system, ^26^^,^^29^ brainstem involvement, ^43^^,^^45^^,^^46^ autoreactivity to antibodies against SARS-CoV-2, ^25^^,^^43^^,^^47^ dyshomeostasis of immune response ^42^^,^^44^ and denervation of peripheral sympathetic nerve fibers. ^26^^,^^43^^,^^42^

This study has some limitations. Due to the small number of cases (especially of POTS patients with COVID-19 infection), generalizability cannot be fully derived. Furthermore, the retrospective collection of data by interview bears the risk of inaccurate symptom recollection and reporting in patients. Finally, effects of mRNA booster vaccinations and other types of vaccination were not recorded in this study.

## Conclusion

The observations of this study suggest that mRNA vaccines are not associated with a higher incidence of acute side effects in patients with POTS and only pose a mild to moderate risk for POTS symptom exacerbation, usually of short duration. POTS symptom exacerbation as a consequence of mRNA vaccination was milder and of shorter duration compared to patients who suffered a COVID-19 infection.

## Ethical statements

### Ethical approval

This study was carried out in accordance with the recommendations of the local ethics committee (Kantonale Ethikkommission Bern, Switzerland, project-ID: 2021-02115; 02.11.2021).

### Consent statement

All subjects gave written informed consent for publication of these data in accordance with the Declaration of Helsinki.

## Data availability

### Underlying data

Dryad: Tolerability of COVID-19 mRNA vaccines in patients with postural tachycardia syndrome https://doi.org/10.5061/dryad.zkh1893bx. ^48^

This project contains the following underlying data:

- Demographic_data.xlsx
- POTS_symptoms_after_COVID-19.xlsx
- POTS_symptoms_after_vaccination.xlsx
- Side_effects_of_mRNA_vaccines.xlsx
- Symptoms_of_COVID-19_infection.xlsx

Data are available under the terms of the Creative Commons Zero “No rights reserved” data waiver (CC0 1.0 Public domain dedication).

### Extended data

Zenodo: Tolerability of COVID-19 mRNA vaccines in patients with postural tachycardia syndrome https://doi.org/10.5281/zenodo.5925527. ^49^

- Informed_Consent_Form.pdf
- Interview_Checklist.pdf

Data are available under the terms of the Creative Commons Attribution 4.0 International license (CC-BY 4.0).
