# Supplementary material for: Tolerability of COVID-19 mRNA vaccines in patients with postural tachycardia syndrome: a cross-sectional study
Source: F1000Res. 2022 Feb 22;11:215. [Version 1] doi: 10.12688/f1000research.109373.1 (PMC10562791; doi:10.12688/f1000research.109373.1)
Supplement: Peer review history

# Reviewer report: Reviewer response for version 1

**DOI:** 10.5256/f1000research.120865.r209338

**Published:** 2023-10-09

**Authors:** Jorge Rodríguez-Capitán

**Affiliations:** 1 Hospital Universitario Virgen de la Victoria, Málaga, Andalusia, Spain

The authors present an interesting study on a specific, well-defined topic for which there is limited prior evidence, and which also represents a real problem in a group of patients: the safety of mRNA COVID-19 vaccines in patients with postural tachycardia syndrome (POTS). Thus, in my opinion, the authors deserve congratulations for this work in which safety data are provided after vaccination in this patient group, something that is crucial to prevent the undesirable effects of a low vaccination rate in this patient population.

 However, the study has some limitations, most of which have been acknowledged by the authors. Firstly, it is a single-center study with a small sample size. Furthermore, a comparison is made between the worsening of symptoms related to POTS after COVID-19 and the worsening observed after vaccination. The conclusion, based on data from a small number of patients (only 5 post-COVID-19 cases), is that the worsening of symptoms after vaccination is of much lower intensity and duration than after COVID-19. In addition, it is suggested that the worsening of POTS symptoms after COVID-19 is of lower intensity and duration in previously vaccinated patients compared to unvaccinated patients, but this assertion can only be based on 3 unvaccinated patients compared to 2 vaccinated patients. This small sample size limits these conclusions, as acknowledged by the authors.

 In any case, the data do show that vaccination was well-tolerated in patients with POTS, and although it cannot be demonstrated that COVID-19 illness after vaccination reduces the degree of worsening of POTS symptoms, the benefits in terms of preventing severe COVID-19 disease outweigh the risk/benefit balance in favor of vaccination in this patient group.

Is the work clearly and accurately presented and does it cite the current literature?

Yes

If applicable, is the statistical analysis and its interpretation appropriate?

Yes

Are all the source data underlying the results available to ensure full reproducibility?

Yes

Is the study design appropriate and is the work technically sound?

Partly

Are the conclusions drawn adequately supported by the results?

Yes

Are sufficient details of methods and analysis provided to allow replication by others?

Yes

Reviewer Expertise:

Postural tachycardia syndrome related to COVID-19 desease or related to mRNA vaccinatio

I confirm that I have read this submission and believe that I have an appropriate level of expertise to confirm that it is of an acceptable scientific standard.

# Reviewer report: Reviewer response for version 1

**DOI:** 10.5256/f1000research.120865.r135527

**Published:** 2022-05-13

**Authors:** Johann Sellner

**Affiliations:** 1 Department of Neurology, Landesklinikum Mistelbach-Gänserndorf, Mistelbach, Austria

This is an important contribution in the wake of vaccination hesitancy among people with neurologic disease. The study provides early scientific evidence that POTS, a condition that may also occur after infections, is not associated with an increased risk of side effects of SARS-CoV-2 vaccination. Importantly, pronounced and prolonged symptom exacerbation was observed with COVID-19 in patients with POTS. This observation should serve as an additional line of argumentation for the safety and efficacy of SARS-CoV-2 vaccination and identifies patients with POTS as a vulnerable cohort for detrimental outcome of COVID19. The only remark from my side relates to the lack of a control group, ideally sex- and age-matched.

Is the work clearly and accurately presented and does it cite the current literature?

Yes

If applicable, is the statistical analysis and its interpretation appropriate?

Yes

Are all the source data underlying the results available to ensure full reproducibility?

Yes

Is the study design appropriate and is the work technically sound?

Yes

Are the conclusions drawn adequately supported by the results?

Yes

Are sufficient details of methods and analysis provided to allow replication by others?

Yes

Reviewer Expertise:

NA

I confirm that I have read this submission and believe that I have an appropriate level of expertise to confirm that it is of an acceptable scientific standard.

# Response

**Published:** 2022-05-16

**Authors:** Belén Rodriguez

**Affiliations:** Inselspital, University Hospital Bern, Switzerland

Thank you for your review. We agree with you that a sex- and age-matched control group would have further improved the power of the study. Due to the extensive data already available on the tolerability of the vaccine in healthy populations, we decided not to include a control group.
